# Clinical value of contralateral breast cancers detected by pre-operative MRI in patients diagnosed with DCIS: a population-based cohort study

**DOI:** 10.1007/s00330-022-09115-5

**Published:** 2022-09-30

**Authors:** Kristien B. I. M. Keymeulen, Sandra M. E. Geurts, Loes F. S. Kooreman, Lucien E. M. Duijm, Sanne Engelen, Sigrid Vanwetswinkel, Ernest Luiten, Sabine Siesling, Adri C. Voogd, Vivianne C. G. Tjan-Heijnen

**Affiliations:** 1 Department of Surgery, Maastricht University Medical Centre, P.O. Box 5800, 6202 AZ Maastricht, the Netherlands; 2 Division Medical Oncology, Maastricht University Medical Centre, GROW, Maastricht University, Maastricht, the Netherlands; 3 Department of Pathology, Maastricht University Medical Centre, GROW, Maastricht University, Maastricht, the Netherlands; 4 Department of Radiology, Canisius Wilhelmina Hospital, Nijmegen, the Netherlands; 5 Department of Radiology and Nuclear Medicine, Maastricht University Medical Centre, Maastricht, the Netherlands; 6 Department of Surgery, Tawam Hospital UAE, UAE University, Abu Dhabi, United Arab Emirates; 7 Department of Research and Development, Netherlands Comprehensive Cancer Organisation (IKNL), Utrecht, the Netherlands; 8 Department of Epidemiology, Maastricht University Medical Centre, Maastricht, the Netherlands

**Keywords:** Carcinoma, intraductal, noninfiltrating, Breast neoplasms, Magnetic resonance imaging, Registries

## Abstract

**Objectives:**

For patients with ductal carcinoma in situ (DCIS), data about the impact of breast MRI at primary diagnosis on the incidence and characteristics of contralateral breast cancers are scarce.

**Methods:**

We selected all 8486 women diagnosed with primary DCIS in the Netherlands in 2011–2015 from the Netherlands Cancer Registry. The synchronous and metachronous detection of contralateral DCIS (cDCIS) and contralateral invasive breast cancer (cIBC) was assessed for patients who received an MRI upon diagnosis (MRI group) and for an age-matched control group without MRI.

**Results:**

Nineteen percent of patients received an MRI, of which 0.8% was diagnosed with synchronous cDCIS and 1.3% with synchronous cIBC not found by mammography. The 5-year cumulative incidence of synchronous plus metachronous cDCIS was higher for the MRI versus age-matched control group (2.0% versus 0.9%, *p* = 0.02) and similar for cIBC (3.5% versus 2.3%, *p* = 0.17). The increased incidence of cDCIS was observed in patients aged < 50 years (sHR = 4.22, 95% CI: 1.19–14.99), but not in patients aged 50–74 years (sHR = 0.89, 95% CI: 0.41–1.93).

**Conclusions:**

MRI at primary DCIS diagnosis detected additional synchronous cDCIS and cIBC, and was associated with a higher rate of metachronous cDCIS without decreasing the rate of metachronous cIBC. This finding was most evident in younger patients.

**Key Points:**

*• Magnetic resonance imaging at primary diagnosis of ductal carcinoma in situ detected an additional synchronous breast lesion in 2.1% of patients.*

*• In patients aged younger than 50 years, the use of pre-operative MRI was associated with a fourfold increase in the incidence of a second contralateral DCIS without decreasing the incidence of metachronous invasive breast cancers up to 5 years after diagnosis.*

*• In patients aged over 50 years, the use of pre-operative MRI did not result in a difference in the incidence of a second contralateral DCIS or metachronous invasive breast cancer.*

## Introduction

In the Netherlands, more than 2000 women are diagnosed with pure ductal carcinoma in situ (DCIS) annually [1]. If completely excised, DCIS has an excellent prognosis with a 10-year breast-cancer-specific survival rate of at least 97% [2–4]. Second invasive events in the same or in the contralateral breast are responsible for the small proportion of patients dying of breast cancer after a former diagnosis and treatment of DCIS [5, 6]. Some of these second breast cancers are already present at the time of diagnosis of the initial DCIS, but are missed because they are not visible on conventional imaging at that moment [5, 7, 8]. Previous studies have shown that magnetic resonance imaging (MRI) predicts the extent of intermediate/high-grade DCIS more accurately and improves the detection of additional malignant lesions in the ipsi- and contralateral breast in comparison with conventional imaging [7, 9, 10]. In a meta-analysis of 19 studies by Houssami et al, MRI at breast cancer diagnosis identified additional tumour foci in the ipsilateral breast in 16% of patients [11]. In another meta-analysis including 3253 patients, MRI found synchronous contralateral malignancies that were not visible on mammography in 4% of patients [12]. Not surprisingly, MRI is increasingly being used during pre-operative workup of patients diagnosed with DCIS and invasive cancer [13–16].

Most of the patients included in the studies mentioned above had an invasive primary breast tumour. For patients with DCIS, data about contralateral cancers detected by MRI at primary diagnosis and the impact of diagnostic MRI use on the detection and stage of contralateral breast cancers during follow-up are scarce. Noteworthily, prior studies showed that adding MRI to mammography in the pre-operative setting of patients diagnosed with DCIS did not lead to a better surgical outcome and increased mastectomy rates [15, 17], making the use of MRI in this patient group debatable. Conversely, one might hypothesise that in patients with primary DCIS, missed invasive breast cancers or even missed DCIS can have a larger impact on prognosis than in patients with primary invasive breast cancer, since prognosis of DCIS is excellent and generally no adjuvant systemic therapy is administered for DCIS.

The aim of this study was therefore to determine if pre-operative MRI, added to conventional imaging in patients primarily diagnosed with pure DCIS, has an impact on the characteristics, risk and timing of contralateral DCIS and invasive breast cancer during follow-up.

## Methods

### Patients and study design

For this population-based retrospective cohort study, all women diagnosed with primary DCIS in 2011–2015 in the Netherlands were identified through the Netherlands Cancer Registry (NCR). Patients were categorised into two groups: those who received a pre-operative MRI after unilateral DCIS diagnosis versus those who did not receive a pre-operative MRI. We excluded patients who received an MRI before the diagnosis of the first primary DCIS. We also excluded patients with a mammography-detected synchronous contralateral breast lesion.

‘Diagnosed with primary DCIS’ was defined as first in time biopsied. DCIS was histologically confirmed using stereotactic, ultrasound- or MRI-guided core needle or vacuum-assisted biopsies, with a preference for the latter.

According to Dutch guidelines, diagnostic imaging comprised full-field digital mammography and ultrasonography in all patients, with breast MRI being considered in patients with high-grade DCIS preferring breast-conserving surgery, those with unclear tumour size, or if there is suspicion of micro-invasion based on the pre-operative biopsy [18].

We obtained follow-up data of contralateral breast lesions and vital status up to and including December 31st, 2019.

### Data collection

Patient, tumour and treatment characteristics were retrieved from the NCR. Patients were included in the NCR database after notification by the nationwide Dutch Pathology Archive of Histo- and Cytopathology on breast cancer diagnosis. Specially trained data managers collected the data from the patients’ files in all Dutch hospitals.

Invasive breast cancers were TNM staged according to the 7^th^ edition of the UICC [19]. Pathological TNM stage was reported, except in the case of neo-adjuvant treatment or unknown pTNM, in which cases clinical TNM stage was used. Invasive breast cancers were categorised as hormone receptor (HR) +/human epidermal growth factor receptor 2 (HER2)− (including HR+/HER2 unknown), HR+/HER2+, HR−/HER2+ and triple negative (TN, i.e. HR−/HER2−) disease.

### Statistical analyses

Contralateral malignant breast lesions were categorised as synchronous (within 3 months after the primary DCIS diagnosis) or metachronous (≥ 3 months after the primary DCIS diagnosis). Analyses were performed separately for the risk of contralateral DCIS and for the risk of contralateral invasive breast cancer. The synchronous and metachronous detection of contralateral DCIS and invasive breast cancer was compared for the patients receiving MRI versus an age-matched control group without MRI. One-to-one matching was performed using 5-year age categories.

The proportion of MRI-detected synchronous contralateral malignant breast lesions was determined by dividing the number of patients with an MRI-detected contralateral breast lesion by the total number of patients with DCIS receiving an MRI.

Five-year overall survival was defined as the time from date of diagnosis to date of death from any cause or censored at last follow-up.

The five-year cumulative incidence of contralateral malignant breast lesions, i.e. synchronous plus metachronous, was calculated using competing risk methods, considering death as competing event, and censoring patients at last follow-up. To determine if MRI at diagnosis resulted in a lower rate of contralateral disease later-on, the cumulative incidence of metachronous malignant lesions for the MRI and age-matched control group was compared using competing risk regression (resulting in a subdistribution hazard ratio (sHR)). Cumulative incidence analyses were stratified by age (below 50 years and aged 50–74 years). In the study period, breast cancer screening was performed biennially with 2-view digital mammography for women aged 50–74 years; for that reason, we used these age limits to define age categories. The number of patients with DCIS of 75 years or older was small, and therefore, these patients were excluded from the age-specific analyses.

Patient, tumour and treatment characteristics of the primary DCIS, and of the synchronous and metachronous contralateral breast lesions for the MRI group, were compared with those of the age-matched control group. Age at primary DCIS diagnosis between these groups was compared by the Mann-Whitney *U* test. Comparison of all other variables was performed using Fisher’s exact test.

## Results

### Baseline characteristics

**Fig. 1:**
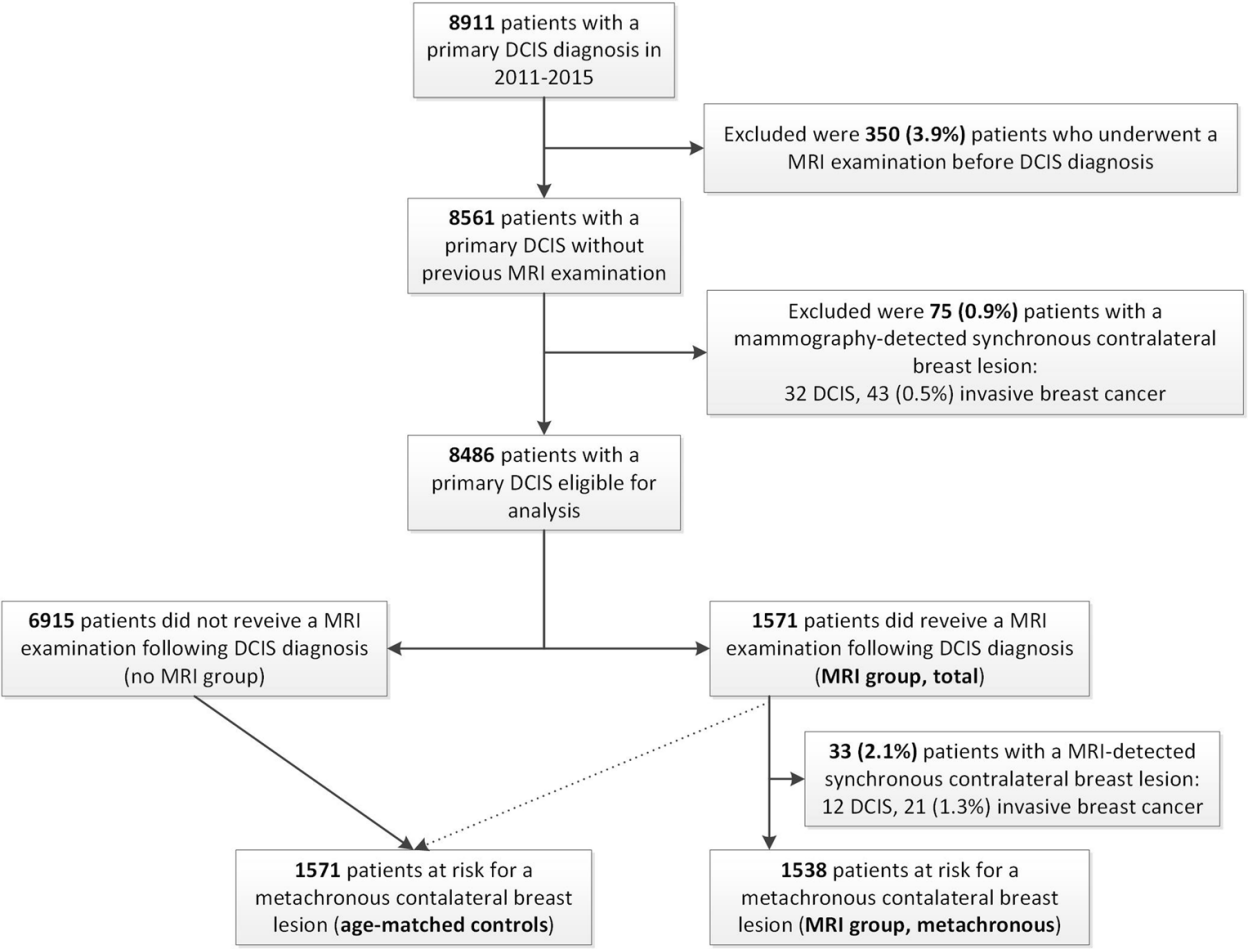
Study flowchart

In the Netherlands, 8911 patients were diagnosed with primary DCIS in 2011–2015. After excluding 350 patients who received an MRI before primary DCIS diagnosis (3.9%) and another 75 (0.9%) patients with a mammography-detected synchronous contralateral (pre)malignant breast lesion, 8486 patients were considered eligible for this study (Fig. 1). Of these, 1571 (19%) received a pre-operative MRI after unilateral DCIS diagnosis. MRI was used in 33% of patients < 50 years, versus 17% of patients 50–74 years and 8% of patients ≥ 75 years of age.

Patients in the MRI group were more often diagnosed with high-grade DCIS and more often underwent mastectomy as compared with the age-matched controls (Table 1). Patterns were similar for patients aged < 50 and 50–74 years, with the highest mastectomy rate in patients aged < 50 years.

**Table 1:** Characteristics of patients primarily diagnosed with ductal carcinoma in situ (DCIS) in 2011–2015: MRI group and age-matched controls by age group

	All ages		< 50 years		50–74 years	
	MRI group	Age-matched controls	MRI group	Age-matched controls	MRI group	Age-matched controls
	(*n* = 1571)	(*n* = 1571)	(*n* = 379)	(*n* = 379)	(*n* = 1165)	(*n* = 1165)
Primary DCIS grade, *n* (%)						
Low	119 (8%)	267 (17%)	26 (7%)	81 (22%)	90 (8%)	180 (15%)
Intermediate	505 (33%)	544 (35%)	129 (35%)	131 (35%)	364 (32%)	401 (35%)
High	927 (60%)	732 (47%)	218 (58%)	161 (43%)	698 (61%)	562 (49%)
Unknown	*n = 20*	*n = 28*	*n = 6*	*n = 6*	*n = 13*	*n = 22*
Surgery*, *n* (%)	1544 (98%)	1520 (97%)	374 (99%)	363 (96%)	1147 (98%)	1134 (97%)
BCS	801 (51%)	1078 (69%)	136 (36%)	218 (58%)	657 (56%)	844 (72%)
Mastectomy	743 (47%)	441 (28%)	238 (63%)	145 (38%)	490 (42%)	289 (25%)
None	27 (2%)	52 (3%)	5 (1%)	16 (4%)	18 (2%)	32 (3%)
Radiotherapy, *n* (%)	738 (47%)	959 (61%)	127 (34%)	193 (51%)	605 (52%)	755 (65%)

Percentages may not add up to 100% because of rounding

*BCS* breast-conserving surgery, *IQR* interquartile range

*Type of surgery missing for one patient aged 50–74 years

### Contralateral DCIS

Median follow-up time was 6.4 years (interquartile range (IQR): 5.1–7.6). Five-year overall survival was 97% (95% CI: 96–98%) for the MRI group and 97% (95% CI: 96–97%) for the age-matched control group. Of the 1571 (19%) patients who underwent an MRI at primary DCIS diagnosis, 12 (0.8%) were diagnosed with a synchronous contralateral DCIS. In patients aged under 50 years, the rate of synchronous contralateral DCIS was 1.3% (5 out of 379), and for patients aged 50–74 years, 0.6% (7 out of 1156).

**Fig. 2:**
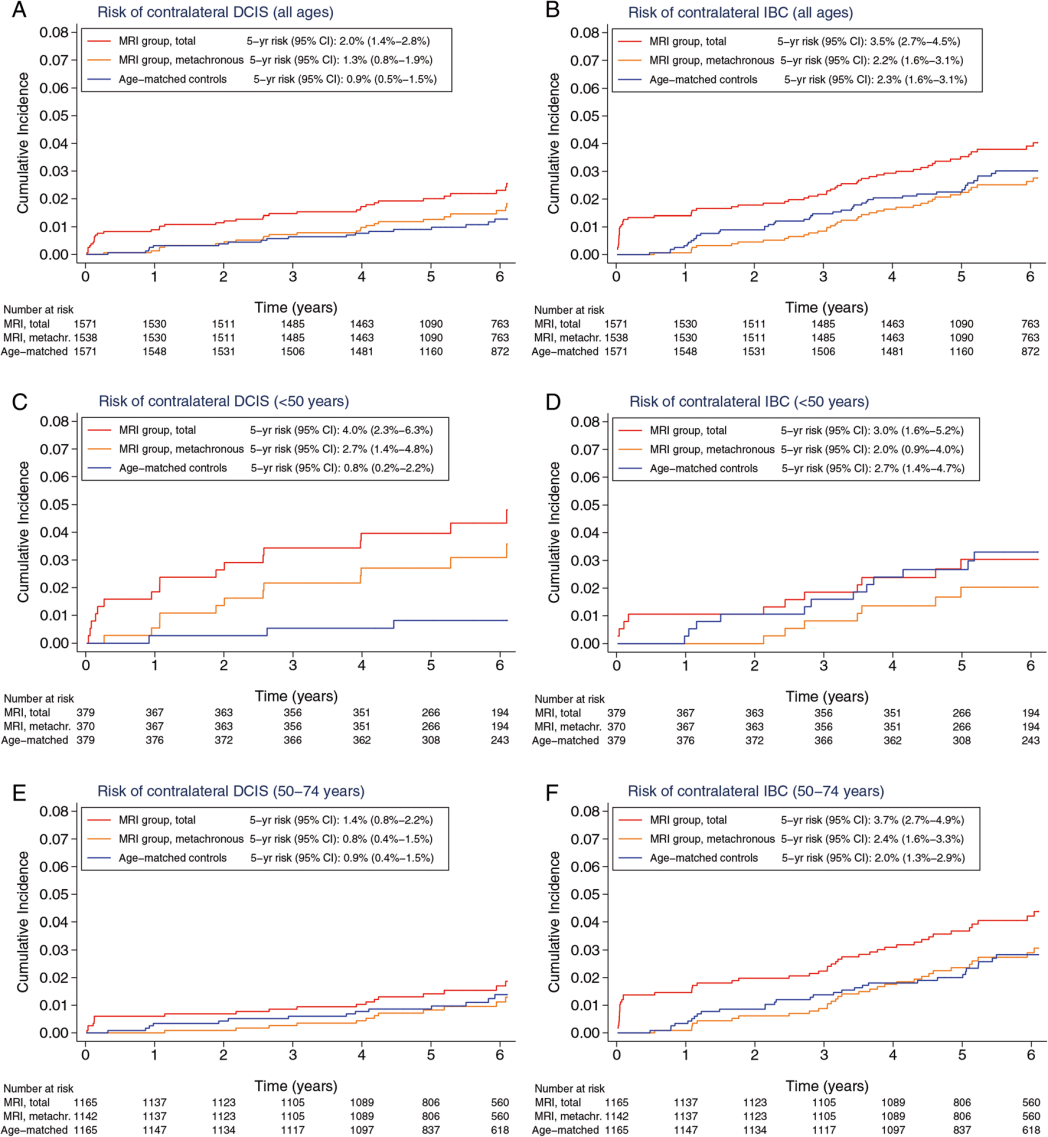
The risk of contralateral ductal carcinoma in situ (DCIS) and contralateral invasive breast cancer (IBC) by use of MRI at primary DCIS diagnosis. Risk of (**A**) contralateral DCIS in all patients, (**B**) contralateral IBC in all patients, (**C**) contralateral DCIS in patients aged < 50 years, (**D**) contralateral IBC in patients aged < 50 years, (**E**) contralateral DCIS in patients aged 50–74 years and (**F**) contralateral IBC in patients aged 50–74 years

The 5-year cumulative incidence of contralateral DCIS (synchronous plus metachronous) was 2.0% (95% confidence interval (CI): 1.4–2.8%) for the MRI group and 0.9% (95% CI: 0.5–1.5%) for the age-matched control group (Fig. 2A). In patients aged under 50 years, the 5-year cumulative incidence of contralateral DCIS was 4.0% (95% CI: 2.3–6.3%) for the MRI group and 0.8% (95% CI: 0.2–2.2%) for the age-matched control group (Fig. 2C). In patients aged 50–74 years, the 5-year cumulative incidence of contralateral DCIS was 1.4% (95% CI: 0.8–2.2%) for the MRI group and 0.9% (95% CI: 0.4–1.5%) for the age-matched control group (Fig. 2E).

In patients aged under 50 years, the cumulative incidence of metachronous contralateral DCIS was statistically significantly higher in the MRI group than in the age-matched controls (sHR = 4.22 (95% CI: 1.19–14.99), *p* = 0.03). In patients aged 50–74, the risk of metachronous contralateral DCIS was not statistically different (sHR = 0.89 (95% CI: 0.41–1.93), *p* = 0.77).

Synchronous contralateral DCIS in the MRI group included more low-intermediate-grade lesions than in metachronous DCIS (91% vs 54%, *p* = 0.06, Table 2). Furthermore, a higher mastectomy rate was observed for synchronous (83%) than metachronous (46%) DCIS (*p* = 0.04). The characteristics of (synchronous plus metachronous) contralateral DCIS detected in the MRI group and age-adjusted controls were comparable.

**Table 2:** Characteristics of patients diagnosed with contralateral ductal carcinoma in situ (DCIS) after primary DCIS by MRI use and moment of detection

	MRI group			MRI group	Age-matched controls	
	Synchronous (*n* = 12)	Metachronous (*n* = 28)	*p* value^$^	Total (*n* = 40)	Total (*n* = 24)	*p* value^†^
Age at diagnosis, median (IQR), years	51 (40–56)	50 (46–58)	0.61	51 (45–57)	53 (49–66)	0.03
Grade, *n* (%)			0.06			0.48
Low	4 (36%)	4 (15%)		8 (22%)	8 (35%)	
Intermediate	6 (55%)	10 (39%)		16 (43%)	7 (30%)	
High	1 (9%)	12 (46%)		13 (35%)	8 (35%)	
Unknown	*n = 1*	*n = 2*		*n = 3*	*n = 1*	
Surgery, *n* (%)	11 (92%)	26 (93%)	1.00	37 (93%)	23 (96%)	0.52
BCS	1 (8%)	13 (46%)		14 (35%)	12 (50%)	
Mastectomy	10 (83%)	13 (46%)	0.04	23 (58%)	11 (46%)	0.21
None	1 (8%)	2 (7%)		3 (8%)	1 (4%)	
Radiotherapy, *n* (%)	1 (8%)	9 (32%)	0.23	10 (25%)	6 (25%)	1.00

*BCS* breast-conserving surgery, *IQR* interquartile range

^$^Comparison of synchronous with metachronous DCIS in the MRI group

^†^Comparison of all contralateral DCIS in the MRI group versus the age-matched controls

### Contralateral invasive breast cancer

Of the patients who received an MRI, 21 (1.3%) were diagnosed with a synchronous contralateral invasive breast cancer. In patients aged under 50 years, the rate was 1.1% (4 out of 379) and for patients aged 50–74 years, it was 1.4% (16 out of 1156).

The 5-year cumulative risk of contralateral invasive breast cancer, including synchronous cancers, was 3.5% (95% CI: 2.7–4.5%) for the MRI group and 2.3% (95% CI: 1.6–3.1%) for the age-matched control group (Fig. 2B). In patients aged under 50 years, the 5-year cumulative incidence of contralateral invasive breast cancer was 3.0% (95% CI: 1.6–5.2%) for the MRI group and 2.7% (95% CI: 1.4–4.7%) for the age-matched control group (Fig. 2D). In patients aged 50–74 years, the 5-year cumulative incidence of contralateral invasive breast cancer was 3.7% (95% CI: 2.7–4.9%) for the MRI group and 2.0% (95% CI: 1.3–2.9%) for the age-matched control group (Fig. 2F).

Overall, the cumulative incidence of metachronous contralateral invasive breast cancer was not different in the MRI group when compared with that of the age-matched controls (sHR = 0.89 (95% CI: 0.58–1.34), *p* = 0.61), irrespective of age (i.e. sHR = 0.60 (95% CI: 0.24–1.53), *p* = 0.29, for women aged under 50 years and sHR = 1.07 (95% CI: 0.65–1.77), *p* = 0.80, for women aged 50–74 years).

Synchronous contralateral invasive breast cancer in the MRI group comprised more often low-grade (53%) tumours than metachronously detected contralateral invasive breast cancers in the MRI group (10%) (Table 3). The characteristics of all contralateral invasive breast cancers in the MRI group were comparable to those of the contralateral invasive breast cancers detected in the age-matched control group (Table 3).

**Table 3:** Tumour and treatment characteristics of contralateral invasive breast cancers after primary DCIS by MRI use and moment of detection, *n* (%)

Characteristics	MRI group			MRI group	Age-matched controls	
	Synchronous (*n* = 21)	Metachronous (*n* = 47)	*p* value^$^	Total (*n* = 68)	Total (*n* = 56)	*p* value^†^
Age at diagnosis, median (IQR) years	59 (52–66)	57 (52–65)	0.64	57 (52–65)	54 (49–62)	0.07
Histology			0.85			0.13
Ductal	14 (67%)	33 (70%)		47 (69%)	40 (71%)	
(Mixed) lobular	4 (19%)	3 (13%)		10 (15%)	13 (23%)	
Other invasive	3 (14%)	8 (17%)		11 (16%)	3 (5%)	
pT stage			0.77			0.35
pT1	15 (71%)	27 (59%)		42 (63%)	39 (70%)	
pT2	5 (24%)	16 (35%)		21 (31%)	12 (21%)	
pT3	1 (5%)	2 (4%)		3 (5%)	5 (9%)	
pT4	0 (0%)	1 (2%)		1 (2%)	0 (0%)	
Unknown	*n = 0*	*n = 1*		*n = 1*	*n = 0*	
Pathological tumour size						
Median (IQR), mm	12 (4–20)	13 (6–25)	0.29	13 (8–23)	12 (7–17)	0.37
< 15 mm	12 (60%)	23 (52%)	0.83	35 (55%)	31 (62%)	0.49
15–30 mm	5 (25%)	11 (25%)		16 (25%)	13 (26%)	
> 30 mm	3 (15%)	10 (23%)		13 (20%)	6 (12%)	
Unknown	*n = 1*	*n = 3*		*n = 4*	*n = 6*	
N stage			1.00			1.00
pN0	15 (75%)	35 (74%)		50 (75%)	42 (75%)	
pN+	5 (25%)	12 (26%)		17 (25%)	14 (25%)	
Unknown	*n = 1*	*n = 0*		*n = 1*	*n = 0*	
M stage			0.30			0.69
M0	21 (100%)	43 (91%)		64 (94%)	54 (96%)	
M1	0 (0%)	4 (9%)		4 (6%)	2 (4%)	
Subtype			0.17			0.28
HR+/HER2−	18 (90%)	33 (73%)		51 (79%)	47 (86%)	
HR+/HER2+	1 (5%)	1 (2%)		2 (3%)	4 (7%)	
HR−/HER2+	1 (5%)	3 (7%)		4 (6%)	1 (2%)	
HR−/HER2− (TN)	0 (0%)	8 (18%)		8 (12%)	3 (9%)	
Unknown	*n = 1*	*n = 2*		*n = 3*	*n = 1*	
Grade			0.002			0.40
Low	10 (53%)	4 (10%)		14 (23%)	15 (28%)	
Intermediate	7 (37%)	25 (61%)		32 (53%)	31 (59%)	
High	2 (10%)	12 (29%)		14 (23%)	7 (13%)	
Unknown	*n = 2*	*n = 6*		*n = 8*	*n = 3*	
Surgery	20 (95%)	43 (91%)	1.00	63 (93%)	54 (96%)	0.46
BCS	8 (38%)	18 (38%)		26 (38%)	21 (37%)	
Mastectomy	12 (57%)	25 (52%)	0.80	37 (54%)	33 (59%)	0.72
None	1 (5%)	4 (9%)		5 (7%)	2 (4%)	
Radiotherapy	10 (48%)	26 (55%)	0.61	36 (53%)	25 (45%)	0.37
Systemic treatment	11 (52%)	30 (64%)	0.43	41 (60%)	32 (57%)	0.86
Chemotherapy	5 (24%)	15 (32%)	0.58	20 (29%)	14 (25%)	0.69
Endocrine therapy	10 (48%)	23 (49%)	1.00	33 (49%)	27 (48%)	1.00
Targeted therapy	2 (10%)	4 (9%)	1.00	6 (9%)	5 (9%)	1.00

Percentages may not add up to 100 because of rounding

*BCS* breast-conserving surgery, *HER2* human epidermal growth factor receptor 2, *HR* hormone receptor, *IQR* interquartile range

^$^Comparison of synchronous with metachronous contralateral invasive breast cancer in the MRI group

^†^Comparison of all contralateral invasive breast cancers in the MRI group versus the age-matched controls

## Discussion

This population-based study included 8486 patients with primary pure DCIS, of whom 19% received an MRI between diagnosis and surgery. MRI detected synchronous DCIS and invasive disease in the contralateral breast in 2.1% of patients, which was not found by mammography. The synchronous invasive tumours detected by MRI were mainly of low to intermediate grade and of the HR+/HER2− subtype. Different than expected, this synchronous detection did not reduce the detection frequency nor tumour stage of metachronous contralateral invasive tumours. In patients aged below 50 years, MRI added to conventional imaging was associated with a persisting higher rate of DCIS in the opposite breast, without influencing the occurrence of invasive breast cancers being detected during the first 5 years of follow-up.

It is well known that MRI is superior to mammography in detecting breast malignancies. Most research was done in patients with primary invasive breast cancer, with MRI being able to detect synchronous contralateral in situ or invasive disease in 2 to 5% of patients [20, 21]. In contrast, data about synchronous contralateral disease found by MRI in patients with primary DCIS is scarce. Wang et al studied 9166 patients aged 67 years or older diagnosed with DCIS between 2004 and 2009 of whom 13.7% received MRI at diagnosis [22]. In this patient group, MRI detected an additional 4% of contralateral synchronous invasive breast cancers and another 4% of contralateral synchronous DCIS. In a study by Hollingsworth not limited by age, MRI found synchronous in situ or invasive carcinoma in the contralateral breast in 5.6% of 285 women diagnosed with primary DCIS between 2003 and 2010, of whom all underwent MRI [7]. Both studies observed more contralateral synchronous breast malignancies than our 2.1%. The reason for this difference is not clear. It might be the result of a different patient population or by the quality of mammography, but it may also be coincidental.

Patients with DCIS have an excellent prognosis, with a 10-year disease-specific survival close to 97% [2–4]. As observed by Dawood et al and Narod et al [5, 6], diagnosis of invasive cancer in the opposite breast as a second event in patients treated for DCIS can have a substantial impact on prognosis. When second events are missed by mammography and left untreated for a longer period of time, the impact might even be greater. It is also known that systemic therapy, and especially hormonal therapy, is able to treat up to 40% of on conventional imaging occult contralateral malignancies adequately [23] making the need to find them debatable. In most countries, adjuvant systemic therapy is not given to patients with DCIS, suggesting that undetected contralateral malignancies will become clinically relevant in time, provided that life expectancy of patients is long enough. However, when we look at the results of our entire study population, MRI between diagnosis of DCIS and surgery increased synchronous detection without lowering the number of metachronous invasive malignancies in the contralateral breast. As Fig. 2 suggests, there might be age-related differences: in patients < 50 years, the number of contralateral invasive cancers after 5 years in the control group equalises the number in the MRI group, suggesting that MRI at diagnosis detects clinically relevant contralateral cancers earlier. In patients aged 50–74 years, there is no difference between the incidences of metachronous contralateral invasive cancers in the MRI versus the control group, suggesting that the MRI-detected synchronous tumours had a higher chance of being over-diagnosed. These age-related differences could be influenced by the differences in follow-up between the two groups. One may expect that earlier diagnosis of contralateral invasive cancer by MRI would lead to a lower tumour stage, but so far, this is not what we observed. When we compared the characteristics of synchronous, contralateral invasive breast cancers found by MRI with those of the metachronous detected in the age-matched controls, we observed no statistically significant differences in tumour size, grade, receptor status or nodal status (Table 3). Treatment regimens were also comparable between the two groups.

MRI at diagnosis detected a higher number of contralateral DCIS, particularly in patients aged under 50 years. In contrast to contralateral invasive cancer, this higher number persisted over time, and was associated with a five times (4.0% versus 0.8%) higher detection of DCIS in the contralateral breast after 5 years as compared with those in the non-MRI group. It might be that, in this patient group, MRI was not only used at baseline but also during follow-up and in this way contributing to this finding. Unfortunately, we have no information on follow-up examinations to test this hypothesis. The use of MRI in routine follow-up is, however, expected to be small, since, according to the Dutch guideline recommendations, only BRCA1/2 mutation carriers have an indication for annual MRI as part of their follow-up routine. Also in this younger patient group, diagnosis of contralateral DCIS will induce over-treatment in some of them, since not all DCIS will lead to invasive disease. But, it is also known that in younger women, a larger proportion of DCIS progresses to invasive breast cancer when compared with older women [24]. So especially in this patient group, prevention of evolution to an invasive stage may reduce the burden of (neo-)adjuvant systemic therapy and/or improve survival in the long run. Longer follow-up is needed to answer this question.

Looking at all results, it seems that the greatest benefit of MRI in patients diagnosed with DCIS and a mammographic occult contralateral invasive breast cancer or DCIS is that it can save in two out of three (2.1%/3.2%) patients who develop contralateral lesions the psychological and physical stress of a second diagnosis and treatment process later in life. In the Netherlands, with a population size of 17 million inhabitants, the annual incidence of DCIS is 2000 (12 per 100,000). If MRI would be offered to all patients with initial diagnosis of unilateral DCIS, and the additional synchronous detection rate of contralateral invasive breast cancer or DCIS by MRI would still be 2.1%, then annually, 42 women would be spared a second diagnosis later in life. However, we have to keep in mind that a part of these tumours might never become clinical and might be over-diagnosed, especially in older patients [25, 26]. A longer follow-up time is awaited to assess if the higher contralateral DCIS detection rate in the young patients eventually will lead to a lower rate of invasive breast cancers and improved survival.

The strength of our study is its large size, with highly reliable data collected by the data clerks of the NCR, with a median follow-up of 77 months. Of course, our study also has certain limitations. Data were collected retrospectively, and information about how patients were selected for additional MRI at diagnosis was not available. We also do not know whether MRI was used during follow-up in a subgroup of patients. We had no information on breast density, genetic predisposition or family history of breast cancer, but we excluded patients who received an MRI before the diagnosis of the primary DCIS, thereby excluding patients who are screened with MRI because of dense breasts or genetic predisposition. The MRI group included a slightly higher rate of high-grade DCIS than the age-matched control group. However, this is not expected to have influenced the results because previous studies found no association between DCIS grade and the risk of a contralateral breast cancer [27, 28]. Finally, follow-up is too short to study the survival impact of the higher rate of contralateral DCIS found in the MRI group, especially in young patients, and of earlier diagnosis of contralateral invasive carcinoma by MRI.

In conclusion, the use of MRI at time of diagnosis in patients diagnosed with pure DCIS on conventional imaging was associated with the detection of twice as many synchronous contralateral invasive breast cancers. In the entire patient group, this did not lead to a decrease in the occurrence of metachronous invasive cancers. MRI was able to detect almost 40% of invasive contralateral breast cancers up to 5 years earlier, reducing the burden of diagnosis later in life especially in patients aged under 50 years, however, without improving tumour stage or tumour characteristics; therefore, the clinical relevance of these finding can be debated. Also, in women aged under 50 years, a higher number of contralateral DCIS was found, persisting over time. Longer follow-up is needed to study the clinical impact of this finding in these younger patients.

## Abbreviations

BCS: Breast-conserving surgery
cDCIS: Contralateral ductal carcinoma in situ
CI: Confidence interval
cIBC: Contralateral invasive breast cancer
DCIS: Ductal carcinoma in situ
HER2: Human epidermal growth factor receptor 2
HR: Hormone receptor
IQR: Interquartile range
MRI: Magnetic resonance imaging
NCR: Netherlands Cancer Registry
sHR: Subdistribution hazard ratio
TN: Triple negative

## Declarations

### Guarantor

The scientific guarantor of this publication is Prof. Vivianne C.G. Tjan-Heijnen, MD, PhD.

### Statistics and biometry

One of the authors has significant statistical expertise.

### Study subjects or cohorts overlap

Some study subjects or cohorts have been previously reported in Br J Surg. 2019;106(11): 1488-1494.doi: 10.1002/bjs.11299.

### Methodology

• retrospective

• observational

• multicentre study
